# Characterization of novel cellobiose 2-epimerases from *Teredinibacter Haidensis* and *Cellvibrio Japonicus*

**DOI:** 10.1186/s12896-026-01184-4

**Published:** 2026-06-18

**Authors:** Yaxian Liu, Sabine Lutz-Wahl, Lutz Fischer

**Affiliations:** Institute of Food Science and Biotechnology, Department of Biotechnology and Enzyme Science, University of Hohenheim, Garbenstr. 25, 70599 Stuttgart, Germany

**Keywords:** Cellobiose 2-epimerase, Epimerization, Isomerization, Lactose, Cold-adaptability

## Abstract

**Background:**

Cellobiose 2-epimerases (CEs) are promising enzymes that catalyze the conversion of lactose to the bioactive disaccharides epilactose and lactulose. Low-temperature (≤8 °C) lactose bioconversion is essential in dairy applications to prevent microbial contamination. Unfortunately, most CEs are not particularly active at these low temperatures. Therefore, this work aims to discover and characterize novel cold-active CEs.

**Results:**

Two novel mesophilic CEs from *Teredinibacter haidensis* (*Th*CE) and *Cellvibrio japonicus* (*Cj*CE) were characterized, which showed both epi- and isomerization activity additionally at low temperatures. *Th*CE showed a maximum epimerization activity (595.4 ± 4.4 nkat/mg protein) at pH 7.5 and 35 °C, while *Cj*CE achieved maximum epimerization activity (887.4 ± 3.9 nkat/mg protein) at pH 9.5 and 40 °C. In addition, *Cj*CE maintained an >80% activity across a wide pH (6–9.5) and temperature range (30–45 °C). Both CEs exhibited significant activity and stability at 8 °C. Specifically, *Th*CE and *Cj*CE retained around 20 and 40% of their epimerization activity at 8 °C and showed half-lives of 96.3 and 72.2 days, respectively. Moreover, *Cj*CE was also found to be capable of catalyzing the isomerization reaction at 8 °C. The isomerization activity of *Th*CE and *Cj*CE increased ~370-fold (70.8 ± 2.7 nkat/mg protein) and ~230-fold (66.1 ± 0.3 nkat/mg protein), respectively, at lactose concentrations up to 600 mM (35 °C for *Th*CE and 40 °C for *Cj*CE). This demonstrates how important it is to conduct investigations of CEs for lactose isomerization to lactulose up to the solubility limit of lactose.

**Conclusions:**

This study identified two new CEs that exhibit interesting catalytic properties for possible application in the generation of disaccharides with prebiotic properties from lactose at low temperatures.

**Supplementary Information:**

The online version contains supplementary material available at 10.1186/s12896-026-01184-4.

## Background

Cellobiose 2-epimerases (CEs; EC 5.1.3.11) belong to the N-acyl-D-glucosamine 2-epimerase (AGE; EC 5.1.3.8) superfamily [1]. They are able to catalyze the interconversion of the glucose moiety at the C2 position to a D-mannose or D-fructose residue at the reducing end of *β*-1,4-linked oligosaccharides, such as cellobiose, lactose, and mannobiose [2, 3]. CEs are the only known enzymes capable of epimerizing *β*-1,4-linked disaccharides among carbohydrate epimerases and isomerases [4–6]. A catalytic attribute of CEs attracting increasing attention is to catalyze the conversion of lactose to two bio-functional derivatives: epilactose (4-O-*β*-galactopyranosyl-D-mannose) and lactulose (4-O-*β*-galactopyranosyl-D-fructose), without the addition of a co-substrate [7, 8]. Both lactulose and epilactose show prebiotic properties that promote the growth of beneficial gut bacteria. Lactulose has been used in clinical practice since 1957 [9–11]. The enzymatic processes catalyzed by CEs are environmentally friendly and do not require any co-substrate, making them particularly attractive for potential industrial applications. Moreover, CEs have garnered increasing interest due to its broad catalytic activity toward various substrates, whereby the highest epimerization activity was reported for the disaccharide mannobiose [12]. Kim et al. [13] and Park et al. [12] found that besides disaccharides, some CEs also exhibited epimerization or isomerization activities toward monosaccharides (e.g. mannose, glucose) and *α*-1,4- or *β*-1,4-linked trisaccharides (e.g. maltotriose, cellotriose, mannotriose). Generally, the epimerization activity of CEs toward all the aforementioned substrates follows the order: disaccharides > trisaccharides >monosaccharides.

The first CE was found in an anaerobic ruminal bacterium, *Ruminococcus albus* 7 (ATCC27210^T^) (*Ra*CE), by Tyler and Leatherwood in early 1967, demonstrating its epimerization activity toward cellobiose [14]. Ito et al. identified the gene of the CE from *R. albus* NE1 in 2008 and showed that the recombinant *Ra*CE was able to catalyze the epimerization of lactose to epilactose [10]. To date, more than 20 different CEs have been characterized [7]. Most of the CEs characterized are from mesophilic bacteria, such as *Ra*CE [3], *Eubacterium cellulosolvens* CE [15], *Bacteroides fragilis* CE [16], *Flavobacterium johnsoniae* (*Fj*CE) [17], and *Cellvibrio vulgaris* (*Cv*CE) [18]. Four CEs from the thermophiles *Caldicellulosiruptor saccharolyticus* (*Cs*CE), *Dictyoglomus turgidum* (*Dt*CE), *Spirochaeta thermophila* (*St*CE), *and Caldicellulosiruptor obsidiansis* were first reported to catalyze the isomerization between keto- and aldo-disaccharides [1]. It was demonstrated subsequently that *Cs*CE and *Dt*CE can catalyze the isomerization reaction at mesophilic or even lower temperatures, but a high activity of the enzyme was required [19]. The research of Kuschel et al. revealed that mesophilic CEs also exhibited isomerization activities toward lactose, resulting in the formation of lactulose [6]. These findings suggest that all of the CEs may possess the potential to catalyze both epimerization and isomerization reactions. Notably, no CE from a psychrophilic microorganism has been characterized to date. The research group of Chen et al. identified two mesophilic CEs from *Roseburia intestinalis* (*Roin*-CE) and *Treponema brennaborense* (*TrBr*-CE) with activities suitable for low temperatures using a genome mining method based on the computational prediction of molecular dynamic simulation [20, 21].

Cold-active enzymes are of great interest in dairy applications due to their economic and environmental advantages, including energy efficiency, microbial growth suppression, and prevention of undesirable heat-induced reactions in food matrices [7, 22]. CEs were reported to be active in milk, enabling the direct production of epilactose and lactulose from lactose. *CsCE,* for example, converted lactose in milk at 8 °C, resulting in 56.7% lactulose (27.5 g/L) and 13.6% epilactose (6.57 g/L) [19]. The CE from *Dysgonomonas gadei* (*Dg*CE) produced epilactose in milk ultrafiltrate at 8 °C with a yield of 29.9% in a batch process [22]. This capability to function efficiently within the common dairy cold chain (≤8 °C) makes cold-active CEs highly attractive for the in situ production of epilactose and lactulose. However, the poor activity of CEs at low temperatures often demands a higher dosage, which increases the process cost. Therefore, it is necessary to discover new CEs that can be applied at low temperatures.

In this study, two novel CEs were discovered in mesophilic aerobic bacteria, *Teredinibacter haidensis* (*Th*CE) and *Cellvibrio japonicus* (*Cj*CE). These were expressed using recombinant technology in *Escherichia coli* BL21(DE3), then purified and partially characterized biochemically. The epimerization and isomerization activities, as well as the substrate selectivity, were investigated for each CE.

## Material and method

### Chemicals and enzyme

All chemicals used were of analytical grade or higher and purchased from Sigma-Aldrich Chemie GmbH (Steinheim, Germany), Merck KgaA (Darmstadt, Germany), Carl Roth GmbH + Co. KG (Karlsruhe, Germany), Fluka Chemie AG (Buchs, Switzerland), and Santa Cruz Biotechnology, Inc. (Heidelberg, Germany). The Q5® High-Fidelity DNA polymerase and the restriction enzymes were obtained from New England BioLabs GmbH (Frankfurt, Germany). The T4 DNA ligase was obtained from Thermo Fisher Scientific (Karlsruhe, Germany). Two protein standards were used for sodium dodecyl sulfate polyacrylamide gel electrophoresis (SDS-PAGE): Precision Plus Protein™ unstained protein marker (10–250 kDa) was obtained from Bio-Rad laboratories GmbH (Feldkirchen, Germany); unstained protein standard (10–200 kDa) was obtained from New England BioLabs GmbH (Frankfurt, Germany). HisTrap^TM^ high performance column (1 mL) was purchased from Cytiva Europe GmbH (Freiburg, Germany). Protein standards (GE Filtration Calibration Kit LMW) used for size-exclusion chromatography (SEC) were purchased from GE Healthcare (Buckinghamshire, UK). Epilactose (30% (w/w)) was produced as described by Kuschel et al. [6].

### Screening of putative CEs and sequence analysis

Two open reading frames (ORFs; shown in Supplementary Table 1) were used as search templates for in silico screening to discover the new CEs. The ORFs were identified through a combined approach involving PCR-based screening with degenerate primers and in silico screening, using metagenomic DNA isolated from vineyard and cave soil samples collected in Esslingen and Sonnenbühl, Germany. A basic local alignment search tool (BLAST) search for *in-silico* screening was performed through three major protein databases: the Universal Protein Resource Knowledgebase (UniProt, https://www.uniprot.org/blast), the National Center for Biotechnology Information (NCBI, https://blast.ncbi.nlm.nih.gov/Blast.cgi), and the Integrated Microbial Genomes & Microbiomes system (https://img.jgi.doe.gov/). The BLAST hits existing commonly in the three databases were selected by analyzing the characteristics of the microorganism origins, such as growth temperature, possible metabolites, and the sequence similarities to the CEs from *Cs*CE (Uniprot entry no. A4XGA6) and *Ra*CE (Uniprot entry no. E6UB41).

### Construction of the expression vectors for CEs

The four CE genes finally selected from *T. haidensis* (*Th*CE, NCBI accession number: WP_075184903.1), *C. japonicus* (*Cj*CE, NCBI accession number: WP_012485926.1), *Simiduia agarivorans* (*Sa*CE, NCBI accession number: WP_015048640.1), and *Paraglaciecola polaris* (*Pp*CE, NCBI accession number: WP_007103104.1) were synthesized for expression in *E. coli* (Life Technologies, Invitrogen, Geneart, Darmstadt, Germany). The codon-optimized gene sequences, along with the corresponding amino acid sequences, are available in the Supplementary Fig. S1 – S4. These four genes were amplified by PCR using the Q5 polymerase (New England Biolabs Inc. Ipswich, USA) to remove the stop codon for the incorporation of the sequence coding for a C-terminal hexahistidine-tag (His_6_-tag). The respective primers are shown in Supplementary Table 2. The PCR conditions were as follows: initial denaturation at 98 °C for 30 s, 30 cycles of denaturation (98 °C, 10 s), annealing for 30 s at different temperatures for the respective CE: 64 °C (*Th*CE), 64 °C (*Cj*CE), 65 °C (*Sa*CE), 68 °C (*Pp*CE), and elongation (72 °C, 30 s), followed by a final elongation (72 °C, 2 min), then cooled at 10 °C. The amplified CE genes were digested by restriction enzymes *Nde*I and *Xho*I, according to the manufacturer’s protocol. The digested target genes were subsequently ligated to the pET-20b(+) vector by T4 DNA ligase, according to the manufacturer’s protocol. The resulting plasmids pET-20b-*Th*CE_His6, pET-20b-*Cj*CE_His6, pET-20b-*Sa*CE_His6, and pET-20b-*Pp*CE_His6 were transformed into *E. coli* XL1 by heat shock transformation. Plasmids were isolated using a GeneJET Plasmid Miniprep Kit (Thermo Fisher Scientific, Schwerte, Germany) and the respective CE genes were sequenced by Eurofins Genomics (Ebersberg, Germany).

### Expression of recombinant CEs

The recombinant plasmids (pET-20b-*Th*CE_His6, pET-20b-*Sa*CE_His6, pET-20b-*Pp*CE_His6, and pET-20b-*Cj*CE_His6) were individually transformed into *E. coli* BL21(DE3) and plated on Luria-Bertani (LB) agar plates (tryptone: 10 g/L, yeast extract: 5 g/L, NaCl: 5 g/L, agar: 15 g/L, pH 7.5) containing 100 μg/mL ampicillin. Single colonies were picked for protein expression. The recombinant *E. coli* cells were initially cultivated in 1 L shaking flasks with 200 mL LB medium (containing 100 μg/mL ampicillin, pH 7.0) at 37 °C and 200 rpm. After reaching the optical density at 600 nm (OD_600 nm_) of 0.8–1.0, isopropyl-*β*-D-thiogalactopyranosid (IPTG, 0.5 mM) was added for induction and the culture was incubated at 30 °C for 5 h. The cells were then harvested by centrifugation (4 °C, 8,000 × g, 10 min) and washed by 0.9% (w/v) NaCl. Thereafter, the cell pellet was used for protein analysis and preliminary activity confirmation. The strains expressing active CEs were further cultivated in 2 L shaking flasks in 400 mL of the same LB medium at 37 °C, 200 rpm. Induction was initiated with the addition of IPTG (0.5 mM) at the OD_600 nm_ of 0.8–1.0. An amount of 10 mL of samples from each cultivation was taken, centrifuged and washed, as described above, at 1, 2, 4, 6, 8, and 21 h after induction. These samples were used for the determination of the OD_600 nm_, protein content, and epimerization activity. Finally, the main cultures were harvested 21 h after induction. After centrifugation and washing, the resulting cell pellets were stored at −20 °C for further analysis.

The CEs showing overexpression in *E. coli* BL21(DE3) were produced in a bioreactor. Therefore, *E. coli* BL21(DE3) harboring plasmid pET-20b-*Th*CE_His6 (*Th*CE) and pET-20b-*Cj*CE_His6 (*Cj*CE) were used. A single colony of the respective recombinant *E. coli* strain was inoculated into 10 mL of 2x yeast extract tryptone (2YT) medium in a culture tube, followed by inoculation at 37 °C,180 rpm for 16 h. The primary preculture was transferred into 40 mL of 2YT medium in a shake flask and cultured at 37 °C, 100 rpm for 10 h. The second preculture was used to inoculate a 500 mL preculture and incubation was done at 37 °C overnight for high-density fermentation. The bioreactor cultivation was done in a Labfors 5 bioreactor (Infors AG, Munich, Germany) using 2YT medium (containing 100 μg/mL ampicillin) at 37 °C, pO_2_ > 30% and pH 7.0 (controlled by 2 M of NaOH and H_3_PO_4_) and a working volume of 5 L. An amount of 500 mL of the preculture was used for inoculation. Additionally, antifoam 204 (Sigma-Aldrich, Darmstadt, Germany) was added to a final concentration of 100 μL/L. The temperature was lowered to 30 °C when an OD_600 nm_ of around 5 was reached, and 0.5 mM IPTG was added for induction. Samples were taken during the cultivation for analysis of the OD_600 nm_, bio dry mass, and epimerization activity. Finally, the cells were harvested by centrifugation (4 °C, 8,000 × g, 10 min) and washed using 0.9% (w/v) NaCl. The final cell pellet was stored at −20 °C.

### Enzyme purification

The cell pellets were suspended in 100 mM sodium phosphate buffer containing 300 mM NaCl at pH 7.0 to a concentration of 30% (w/v) to purify the *Th*CE and *Cj*CE. The cells were disrupted by ultrasonication (10 cycles containing 1 min disruption, 1 min break (UP200S/S3, Hielscher Ultrasonics, Teltow, Germany)) on ice. After the centrifugation (8,000 × g, 10 min, 4 °C), the supernatant (10 mL) was filtered (0.45 μm) and used for purification by immobilized metal affinity chromatography (IMAC). The supernatant was applied to the HisTrap^TM^ HP column, which was equilibrated with binding buffer (20 mM sodium phosphate buffer, 300 mM NaCl, and 20 mM imidazole, pH 7.5). The unbound protein was washed out with 5 column volume (CV) of binding buffer at 1.0 mL/min, followed by an elution of bound protein with a linear gradient of imidazole from 0 to 250 mM (20 CV) at 1.0 mL/min. The active fractions were collected and the buffer was exchanged to 50 mM sodium phosphate buffer containing 50 mM NaCl (pH 7.0) using a PD-10 column (GE Healthcare, Freiburg, Germany). The resulting solutions were used for further analysis.

### Protein analysis

The protein content was determined according to Bradford [21] using bovine serum albumin as the standard. The protein expression and purification were evaluated by SDS-PAGE using 10% (w/v) acrylamide gel. The visualization of the gel was done via Coomassie Brilliant Blue staining, according to Fairbanks et al. [23]. Two unstained protein standards (10–250 and 10–20 kDa) were used. The molecular weights of the CEs were determined by SEC using the column “Superdex 75 10/300 GL” (GE Healthcare, Freiburg, Germany) on the Äkta FPLC system. Accordingly, the purified CE (500 μL) was applied to the column and eluted by 50 mM sodium phosphate buffer containing 150 mM NaCl (pH 7.0) at 0.8 mL/min. The epimerization activity of the eluted fractions was measured using the substrate lactose. A calibration curve was prepared using the protein standards ribonuclease (13.7 kDa), carbonic anhydrase (29 kDa), ovalbumin (44 kDa), and conalbumin (75 kDa).

### Enzyme assay

The standard assay of CE activity was performed at 37 °C, if not otherwise mentioned. A stock solution of anhydrous lactose (300 mM) was prepared in 50 mM sodium phosphate buffer containing 50 mM NaCl (pH 7.0). The lactose and enzyme solutions were preincubated separately at 37 °C for 5 min. The reaction was started by mixing equal volumes (50 μL) of the enzyme and lactose stock solution. After 10 min incubation, the reaction was stopped by adding 1 M HCl, cooled on ice for 10 min, and then centrifuged at 20,000 × g, 4 °C for 5 min. The supernatant of 100 μL was neutralized with 2 M Tris until it reached pH 7.0. In addition, 10 mM trehalose (120 μL in ddH_2_O) was added to the sample as an internal standard. The sample, after a 1:5 dilution with ddH_2_O, was analyzed by high-performance liquid chromatography (HPLC) for sugar quantification, following the parameters described in Section “High-performance liquid chromatography”. The calibration curves of lactose, epilactose, and lactulose were prepared and the total sugar concentration in each standard sample was set to 150 mM (in ddH_2_O). One unit of epimerization or isomerization activity was defined as the amount of enzyme required to release one μmol of epilactose or lactulose from lactose per minute.

### High-performance liquid chromatography

The HPLC system Agilent 1100 Series (Agilent Technologies, Santa Clara, United States) with a SEDERE LT-ELSD SEDEX85 detector (Sedere, Alfort ville Cedex, France) was used to determine the sugar concentration. The sugars were separated on the Shodex HILICpak VG-50-4E column (4.6 × 250 mm, 5 μm, Shodex, Tokyo, Japan). The temperature of the column and detector was set to 40 and 50 °C, respectively. An amount of 5 μL from the sample was injected into the system, followed by a linear gradient elution of the solvents acetonitrile (A), methanol (B), and ddH_2_O (C) at a flow rate of 0.75 mL/min. The gradient was as follows: 75% A/15% B/10% C (v/v/v, (0 min)), 65% A/15% B/20% C (1–13 min), 50% A/15% B/35% C (13–15 min), 50% A/15% B/35% C (15–17 min), 75% A/15% B/10% C (17–17.5 min), and 75% A/15% B/10% C (17.5–25 min). The calibration range of lactulose, epilactose, and lactose was 2–140, 0.9–43.5, and 7–147.1 mM, respectively.

### Biochemical characterization of recombinant *Th*CE and *Cj*CE

#### Effect of pH and temperature

The partially purified CEs (*Th*CE and *Cj*CE) were biochemically characterized. The pH profile was investigated using purified CEs which were buffer-exchanged to ddH_2_O and diluted with the respective buffer. The buffers used in the assay were as follows (each at 50 mM): sodium acetate (pH 5.0–6.0), sodium phosphate (pH 6.0–7.5), Britton-Robinson (pH 7.5–10.0), Tris-HCl (pH 7.5–9.0), and sodium carbonate (pH 8.5–10.0). The determination of epimerization activity was done at 37 °C.

The influence of the temperature (8–60 °C) on the epimerization activity was investigated in sodium phosphate buffer (50 mM, pH 7.5) for *Th*CE and Tris-HCl buffer (50 mM, pH 9.0) for *Cj*CE. The temperature stability of *Th*CE was investigated at 8, 25, and 35 °C in sodium phosphate buffer (50 mM, pH 7.5). For *Cj*CE, the investigation was done at temperatures of 8, 30, and 40 °C in Tris-HCl buffer (50 mM, pH 9.0). Herein, the protein content of both CEs was standardized to 0.255 g_protein_/L_assay_. The residual epimerization activity was determined at 35 °C for *Th*CE and 40 °C for *Cj*CE. The natural log value of the residual epimerization activity divided by the initial activity (Ln (E_t_/E0)) was plotted against the incubation time (h) for the calculation of the half-life (t_1/2_). The slope of the linear fit curve corresponds to the inactivation constant (*K*_in_). The half-life (t_1/2_) was calculated according to the following equation (Eq. 1):

1 $${{\rm{t}}_{{\rm{1/2}}}}{\rm{ = }}{{{\rm{ln(2)}}} \over {{{\rm{K}}_{{\rm{in}}}}}}$$

#### Michaelis constant determination

The Michaelis constant (*K*_m_) was determined at the optimal pH and maximum temperature of the respective CEs. The determination was done in sodium phosphate buffer (50 mM, pH 7.5) at 35 °C for *Th*CE, and in Tris-HCl buffer (50 mM, pH 9.0) at 40 °C for *Cj*CE. The substrate lactose (anhydrous) concentration varied from 25 to 600 mM. A nonlinear regression method was used to fit the Michaelis-Menten equation for the final calculation of the *K*_m_.

#### Specific epimerization and isomerization activities

The isomerization activities, using 150, 400, and 600 mM lactose as the substrate were determined for *Th*CE and *Cj*CE at their optimum pH and maximum temperature. In addition, the epimerization and isomerization activity at 8 °C and optimal pH were determined for both CEs to evaluate the possibility of epilactose/lactulose production from milk lactose in the dairy industry. All assays were done as described in Chapter 2.7, with an adjusted incubation time. The epilactose or lactulose concentration of the reaction products was quantified by HPLC analysis to finally calculate the activity of epimerization or isomerization.

#### Investigation of the substrate selectivity

To investigate the substrate selectivity of *Th*CE and *Cj*CE, the biotransformations were done using different sugars: glucose, fructose, mannose, lactose, epilactose, lactulose, cellobiose, and maltose. Furthermore, N-acetylglucosamine was also tested as a substrate for the conversion, with the addition of 1 nmol ATP as a co-substrate. The stock solutions of substrate (300 mM) were prepared using the ten substances mentioned previously dissolved in two different buffers (both at 50 mM): sodium phosphate buffer (pH 7.5) was used for the *Th*CE activity assay and Tris-HCl buffer (pH 9.0) for the *Cj*CE activity assay. The substrate solutions (50 μL) and the enzyme (50 μL) were mixed and incubated at 10 °C below the maximum temperature of the respective CE (25 °C for *Th*CE and 35 °C for *Cj*CE). After incubation for 48 h, the reaction was stopped by the addition of 200 μL of 1 M HCl, followed by neutralization to pH 7.0 using 2 M Tris. Thereafter, trehalose (1 mM) was added as the internal standard and then diluted 1:3 with ddH_2_O and used for the HPLC analysis. In addition, the calibration curves of glucose, mannose, and maltose were prepared, and the standards were treated equivalently to the reaction sample (calibration range: 10–150 mM).

### Statistical analysis

Enzyme assays were done at least in duplicate and protein determinations were done in triplicate. Data is presented as mean ± standard deviation. Data analysis and figure plotting were done using Excel (Microsoft Corp., Redmond, USA) or OriginPro 2016 (OriginLab, Massachusetts, USA).

## Results and discussion

### Selection of putative CEs and confirmation of the epimerization activity

Two ORFs (shown in Supplementary Table 1) of CE-like fragments were used as templates for a BLAST search through protein databases to identify novel cold-active CE genes. These CE-like fragments were identified through a combination of PCR-based and in silico screening of metagenomic DNA extracted from vineyard and cave soil samples collected in Esslingen and Sonnenbühl, Germany. The BLAST hits with around 50% identity to the two templates (Supplementary Table 1) were randomly chosen for refinement. The CEs of mesophilic or psychrophilic sources which are active at low temperatures (≤8 °C) are of great interest since they provide significant advantages for applications in the dairy industry [7]. The microbial sources of the putative CEs were initially selected based on the following criteria: a low growth temperature and similar potential metabolic substrates to those utilized by AGE enzymes (e.g. N-acetylglucosamine, cellobiose, and lactose). Additionally, moderate sequence similarities to *Cs*CE (the enzyme with the highest isomerization activity so far) and *Ra*CE (a mesophilic CE with a similar reaction progression to *Cs*CE) were employed as criteria for further mining of the protein database. Four uncharacterized CE-like proteins were ultimately selected: *Th*CE (NCBI accession number: WP_075184903.1), *Cj*CE (NCBI accession number: WP_012485926.1), *Pp*CE (NCBI accession number: WP_007103104.1), and *Sa*CE (NCBI accession number: WP_015048640.1). These putative CE sequences all contained the catalytic residues corresponding to His184, His243, and His374 of *Ra*CE [24]. The four putative CEs showed identity of around 50% to each other and similarity of 30–40% to *Cs*CE or *Ra*CE. In contrast to *C. saccharolyticus* and *R. albus* (anaerobic, Gram-positive bacteria), the four microorganisms were aerobic, Gram-negative bacteria. Among them, *P. polaris* is a psychrophile that grows at temperatures of 5–30 °C, and *T. haidensis*, *C. japonicus* and *S. agarivorans* are mesophiles with a growth temperature range of 15 to 50 °C.

The genes encoding the putative CEs (*Th*CE, *Cj*CE, *Pp*CE, and *Sa*CE) were synthesized in vitro and cloned into the expression plasmid pET20b for recombinant expression in *E. coli* BL21(DE3). Initial shake flask cultivation trials of the recombinant strains on a 200 mL scale resulted in the overexpression of *Th*CE and *Cj*CE. After induction for 5 h, the crude extracts of *Th*CE and *Cj*CE exhibited distinct bands at around 40 kDa on SDS-PAGE, whereas *Pp*CE and *Sa*CE only displayed very faint bands (Supplementary Fig. S5). Additionally, epimerization activity toward lactose was detected in the crude cell extracts of *Th*CE and *Cj*CE, but no activity was observed for *Pp*CE or *Sa*CE (data not shown). The results indicated the successful heterologous expression of *Th*CE and *Cj*CE in *E. coli* BL21(DE3). Therefore, the putative CEs of *Th*CE and *Cj*CE were analyzed in more detail.

### Recombinant production of *Th*CE and *Cj*CE in *E. coli*

**Fig. 1:**
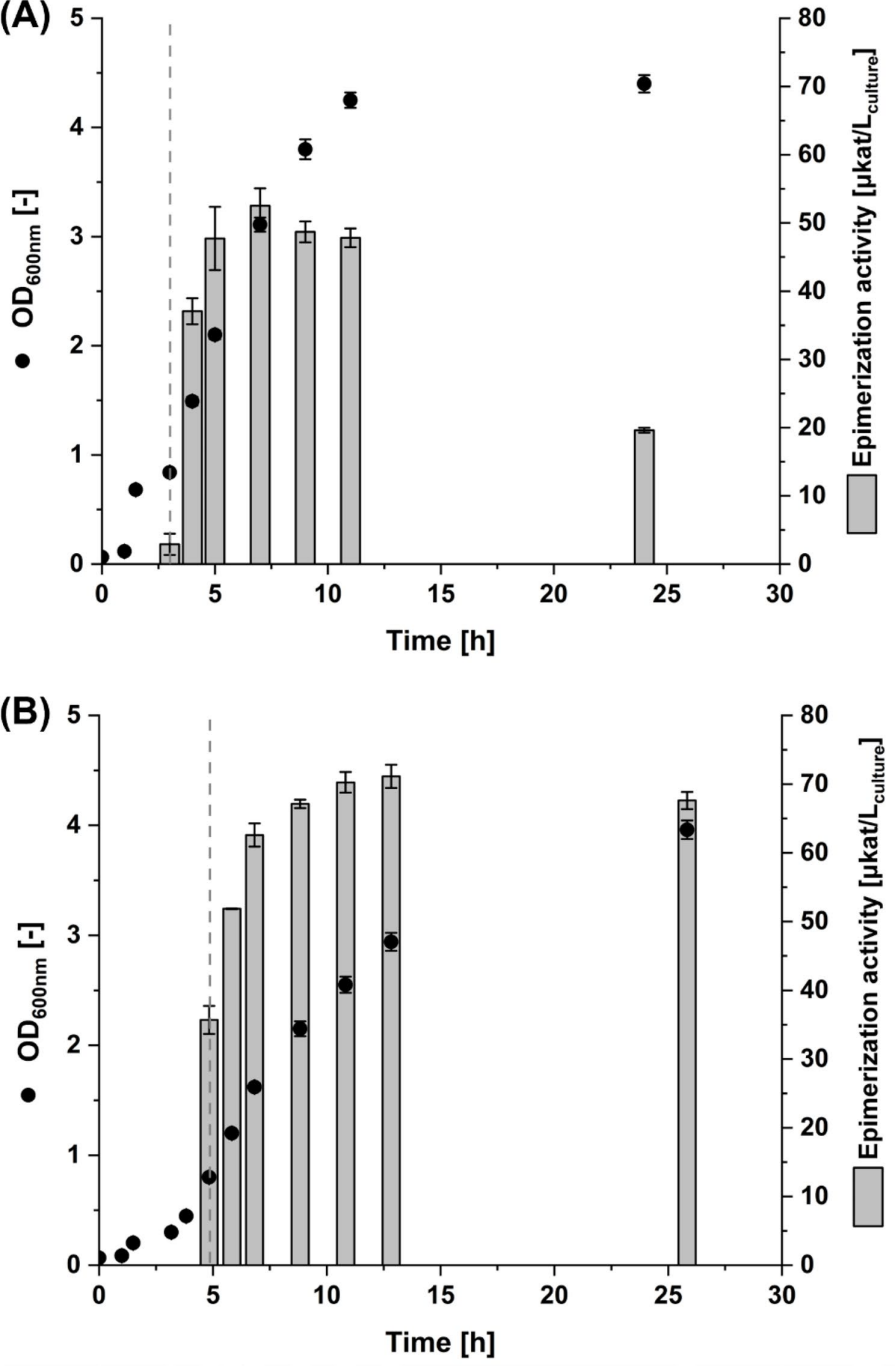
Shake flask cultivation of recombinant *E. coli* BL21(DE3) for the production of the cellobiose 2-epimerases (CEs). (**A**) *Teredinibacter haidensis* (*Th*CE), (**B**) *Cellvibrio japonicus* CE (*Cj*CE). Working Vol. 400 mL. The dashed line indicates induction with IPTG (0.5 mM) and a temperature shift from 37 to 30 °C

After the initial cultivation on a 200 mL scale, the cultivation of *E. coli* BL21(DE3) harboring the plasmids pET20b-*Th*CE-His6 (*Th*CE) and pET20b-*Cj*CE-His6 (*Cj*CE) was done in shake flasks using 400 mL LB medium, respectively. As a control, *E. coli* BL21(DE3) containing the empty vector pET-20b(+) was used to eliminate interference from host basal expression. For *Th*CE an OD of 4.4 was obtained at the end of the cultivation (24 h), as shown in Fig. 1A. The highest epimerization activity of 52.5 ± 2.6 μkat/L_culture_ was obtained after induction for 4 h. For *Cj*CE an OD of 4.0 was reached at the end of the cultivation (26 h). After induction for 8 h, the *Cj*CE epimerization activity reached its maximum value of 71.1 ± 1.7 μkat/L_culture_ (Fig. 1B). As expected, no epimerization activity was observed in the cell extract of the control strain. The epimerization activities we observed were similar to those reported by Ojima et al. [25] (87.5 μkat/L culture), who used the *Rhodothermus marinus* CE produced via recombinant technology in *E. coli* BL21(DE3). Bioreactor cultivations were performed using 2YT medium with a working volume of 5 L to produce *Th*CE and *Cj*CE on a larger scale for further characterization. The highest *Th*CE activity of 100.6 ± 3.0 μkat/L_culture_ was achieved after cultivation for 4.4 h, as illustrated in the Supplementary Fig. S6A. By the end of cultivation (6.7 h), the sum of the total *Th*CE activity was 373.0 μkat. The maximal *Cj*CE activity of 438.4 ± 6.2 μkat/L_culture_ was measured at the end of the cultivation (12 h), resulting in the sum of the total *Cj*CE activity of 2126.0 μkat (see Supplementary Fig. S6B). The production levels of both CEs increased compared to shake flask cultivation. *Th*CE showed a 2-fold increase, while *Cj*CE demonstrated a 6-fold increase in epimerization activity. The production levels of CEs achieved in this study were higher than those of *D. gadej* CE in *E coli* Rosetta-gami (DE3) (49.0 µkat total epimerization activity) [22] and *Cs*CE in *E. coli* BL21(DE3) (27.7 µkat total epimerization activity) [19]. However, these levels are lower than those of *Fj*CE in *E. coli* BL21(DE3), which resulted in a total activity of 1.14 kat [26].

### Purification of *Th*CE and *Cj*CE

**Fig. 2:**
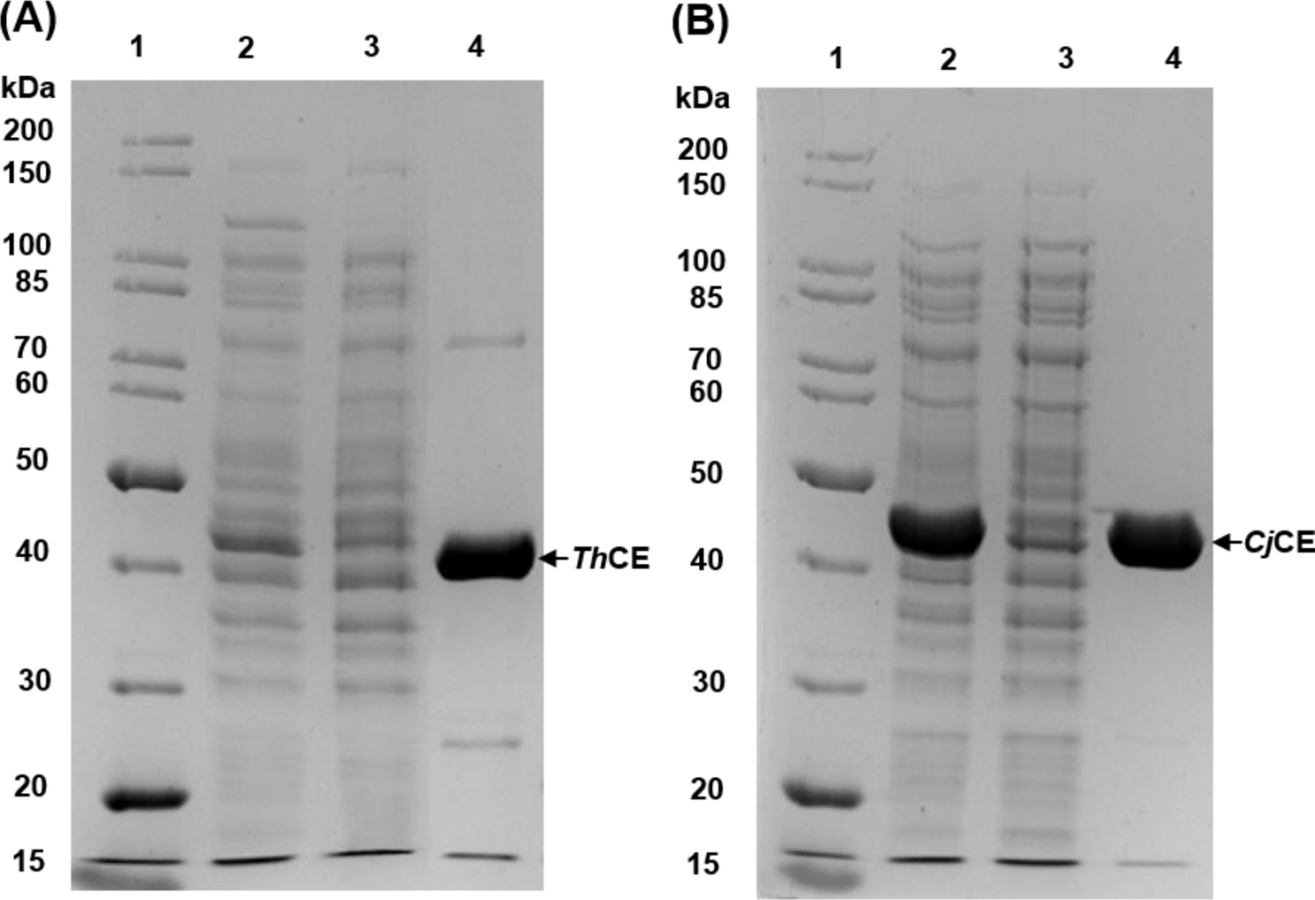
Sodium dodecyl sulfate polyacrylamide gel electrophoresis (SDS-PAGE) analysis of the purification of *Th*CE (**A**) and *Cj*CE (**B**). Lane 1: unstained protein standard (10–200 kDa, New England BioLabs GmbH), lane 2: crude extract, lane 3: flow through during immobilized metal affinity chromatography (IMAC), lane 4: purified CE (IMAC)

In order to purify *Th*CE and *Cj*CE from bioreactor cultivations, IMAC was used on the supernatant after cell disruption. After IMAC purification, *Th*CE and *Cj*CE were purified 12.1- and 3.0-fold, respectively, as shown in Table 1. The specific activities reached 380.9 nkat/mg protein for *Th*CE and 140.4 nkat/mg protein for *Cj*CE, with activity yields of 74.1 and 89.0%, respectively. The higher purification factor of *Th*CE compared to *Cj*CE may be due to the former being generated at a lower rate during the cultivation step. The higher purification factor of *Th*CE compared to *Cj*CE might be due to its lower expression during the cultivation step. This assumption can be verified by the result of the SDS-PAGE analysis of the purification (Fig. 2). It is notable that the crude extract of *Cj*CE showed a stronger band than *Th*CE from the same content of protein (5 μg) at about 40–50 kDa, which corresponds to the theoretically calculated molecular weight of 47.0 and 49.1 kDa for *Th*CE and *Cj*CE (Table 2), respectively, according to their amino acid sequences (*Th*CE = 398 aa, *Cj*CE = 415 aa).

**Table 1:** Purification of the recombinant *Th*CE and *Cj*CE by IMAC

		Total protein	Total Activity	Specific activity	Yield	Purification factor
		[mg]	[nkat]	[nkat/mg]	[%]	[-]
*Th*CE	Crude extract	78.4	2463.1	31.4	100	1
	IMAC	4.8	1825.6	380.9	74.1	12.1
*Cj*CE	Crude extract	165.5	8160.9	46.9	100	1
	IMAC	51.7	7263.6	140.4	89.0	3

**Table 2:** Biochemical properties of the CEs (*Th*CE and *Cj*CE).

	*Th*CE	*Cj*CE
Molecular weight [kDa]		
Theoretical^a^	47.0	49.1
Size-exclusion chromatography (SEC)	43.9	41.2
pH optimum [-]	7.5	7.0–9.5
Temperature-maximum [°C]	35	40
*K*_m_^b^ [mM]	247.3	524.0

^a^ Theoretical value of molecular weight was calculated from the amino acid sequences of the CEs

^b^ Determination was done at pH optimum and temperature maximum with lactose (25–600 mM)

**Fig. 3:**
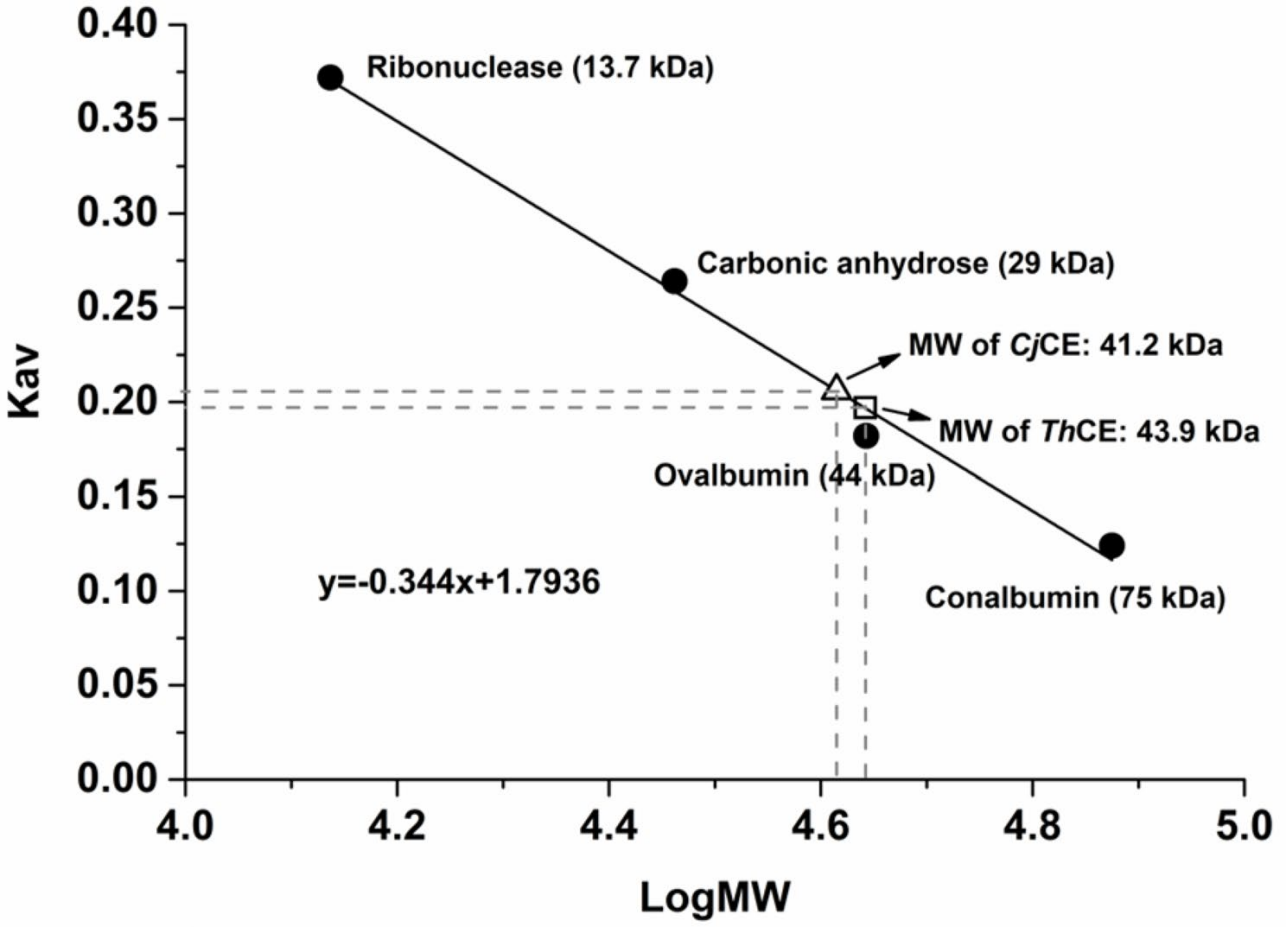
Molecular weight determination of the CEs by size-exclusion chromatography (SEC). Protein standards (ribonuclease: 13.7 kDa, carbonic anhydrase: 29 kDa, ovalbumin: 44 kDa, and conalbumin: 75 kDa) were used for the calibration curve

In addition to SDS-PAGE, the molecular weight of *Th*CE and *Cj*CE was further determined using SEC, resulting in a calculated molecular weight of 43.9 and 41.2 kDa for *Th*CE and *Cj*CE, respectively (Fig. 3 and Table 2). This correlates with the results of the SDS PAGE showing one single band between 37 and 50 kDa (Supplementary Fig. S7). These results indicate that both CEs are present as monomers, which aligns with other reported CEs that have a molecular weight within the 40–50 kDa range [1]. Additionally, SEC results have identified many other CEs, such as *Cs*CE [27], *Dt*CE [13], and *St*CE [12], as monomeric proteins.

### Effect of pH and temperature on the activity of *Th*CE and *Cj*CE

**Fig. 4:**
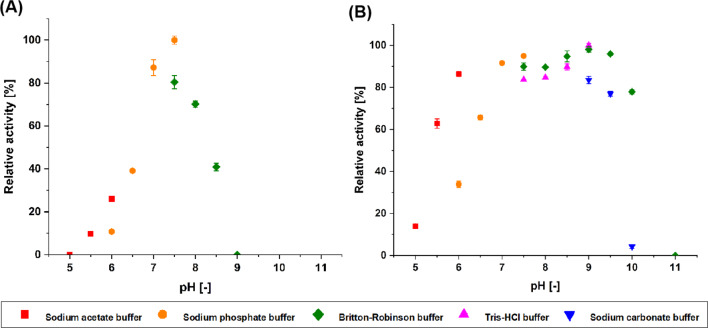
The influence of pH on the epimerization activity of the CEs (37 °C). (**A**) *Th*CE (100% = 336.59 nkat/mL), (**B**) *Cj*CE (100% = 1272.42 nkat/mL)

The pH and temperature profiles of the purified *Th*CE and *Cj*CE were investigated by measuring the epimerization activity using lactose as the substrate. The optimum pH for *Th*CE was found to be 7.5, whereas *Cj*CE maintained a broad pH profile, retaining ≥ 80% of its maximum activity (observed at pH 9.0 in Tris-HCl buffer) from pH 7.0 to 9.5 (Fig. 4 and Table 2). Consequently, a pH of 9 was chosen for *Cj*CE for further experiments. *Th*CE, showed more than 80% of its relative activity at pH 7, but displayed complete inactivation at pH 5.0 and pH 9.0 (Fig. 4A). By contrast, *Cj*CE exhibited a broad pH range, with relative activity above 80% from pH 6.0 to 9.5, and retained about 15% of its relative activity at pH 5.0. However, it was completely inactivated at pH 11.0. The inactivation of both CEs at extremely acidic or basic conditions could be due to the damage of their three-dimensional structure, especially the active sites, which disrupts substrate binding and catalytic function [28, 29]. Such a broad pH range as *Cj*CE was commonly observed for several other CEs, including *Dt*CE [30], *Roin*-CE [20], and *TrBr*-CE [31].

Both *Th*CE and *Cj*CE exhibited various activities at the same pH in different buffers. The epimerization activity was higher in sodium acetate buffer than in sodium phosphate buffer at pH 6.0 for both enzymes. Additionally, high relative activity (around 80%) was determined in Britton-Robinson buffer, whereas hardly any activity (around 5%) was measured in sodium carbonate buffer at pH 10.0 (Fig. 4). This suggests that the activity of the two CEs may depend on the salt composition. This phenomenon has also been observed in many other CEs, as reported for *Ra*CE [10], *St*CE [12], *Roin*-CE [20], *Trbr*-CE [31], and *Dt*CE [30].

**Fig. 5:**
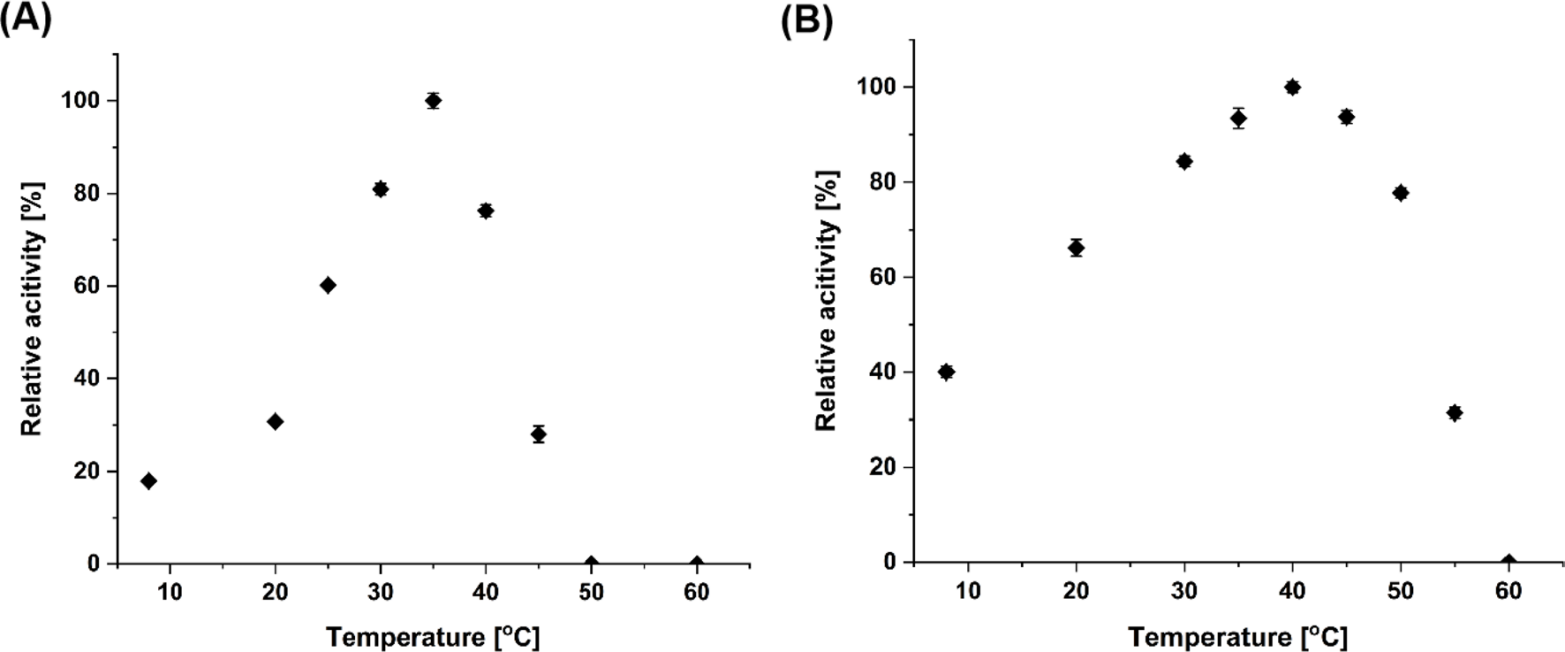
The influence of temperature on the epimerization activity of the CEs. (**A**) *Th*CE (100% = 300.84 nkat/mL), (**B**) *Cj*CE (100% = 1178.83 nkat/mL). The epimerization activity of *Th*CE was measured in sodium phosphate buffer (50 mM, pH 7.5). The epimerization activity of *Cj*CE was measured in Tris-HCl buffer (50 mM, pH 9.0)

Figures 5A and B show the temperature profiles of *Th*CE and *Cj*CE, respectively. The maximum temperature for *Th*CE was 35 and 40 °C for *Cj*CE, as summarized in Table 2. This correlates with the maximum temperatures reported for other CEs: for example, 35 °C for *Fj*CE [6], and 40 °C for both *Dg*CE and *Cellulosilyticum lentocellum* CE [22]. The epimerization activity dropped dramatically for *Th*CE, from around 80 to 30%, when the temperature increased from 40 to 45 °C. By comparison, *Cj*CE exhibited a broader temperature range maintaining over 80% of its residual activity between 30 and 45 °C. No activity of *Th*CE and *Cj*CE was detected at 50 and 60 °C, respectively. At 8 °C, *Th*CE retained around 20% of its relative activity, whereas *Cj*CE retained around 40%. These results indicated that both of the CEs exhibited certain low-temperature adaptability, with *Cj*CE demonstrating even greater adaptability than *Th*CE. This was consistent with reports on other cold-active, mesophilic CEs, which maintained considerable relative activity at 8 °C: approximately 30% for *T. brennaborense* CE [31], 37% for *R. intestinalis* CE [20], and 53% for *R. albus* NE1 CE [10]. The cold-adaptability observed could be attributed to the high structural flexibility of the CEs, as noted in the study of Chen et al. [20].

### Temperature stability of *Th*CE and *Cj*CE

**Fig. 6:**
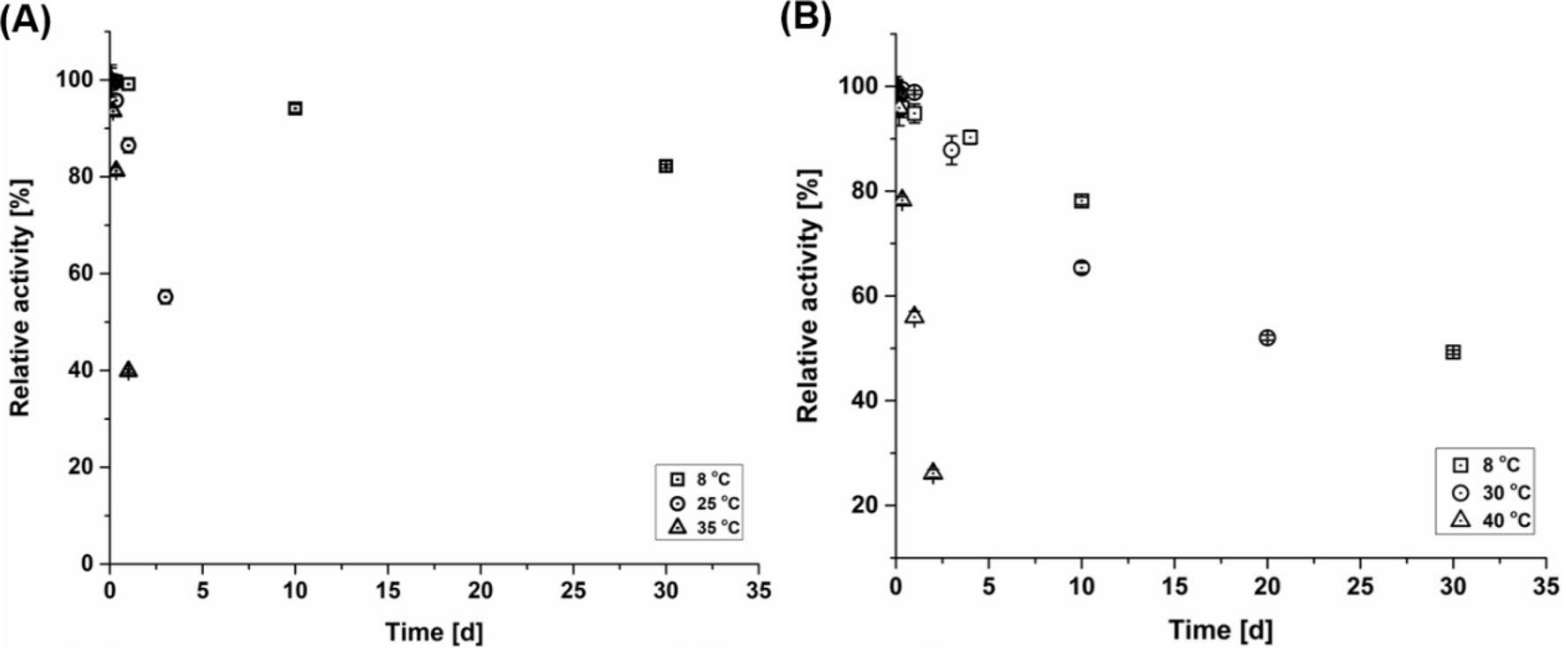
The temperature stability of the CEs. (**A**) *Th*CE (100% = 108.73 nkat/mL), (**B**) *Cj*CE (100% = 174.59 nkat/mL). The stability of *Th*CE was investigated at 8, 25, and 35 °C in sodium phosphate buffer (50 mM, pH 7.5) and the epimerization activity was measured at 35 °C. The stability of *Cj*CE was investigated at 8, 30, and 40 °C in Tris-HCl buffer (50 mM, pH 9.0) and the epimerization activity was measured at 40 °C

The temperature stability of *Th*CE and *Cj*CE was investigated at 8 °C, at the respective temperature maximum, and 10 °C below their temperature-maximum (Fig. 6). The stability of *Th*CE and *Cj*CE was relatively poor at their respective maximum temperatures of 35 and 40 °C, with 35 and 56% of their initial activity remaining after a one-day incubation, respectively. This trend is consistent with that of most reported mesophilic CEs, such as those from *D. turgidum* ATCC 700827, *Teredinibacter turnerae* ATCC 39867, and *F. johnsoniae* NBRC 14942 [17]. Both *Th*CE and *Cj*CE retained > 85% relative activity after a one-day incubation at temperatures 10 °C below their maximums, demonstrating their enhanced stability. After 30 days of incubation, *Th*CE retained 82% of its initial activity at 8 °C, while *Cj*CE retained only 49%.

Similarly, the half-lives of *Th*CE and *Cj*CE (Table 3) were calculated according to the inactivation rate constant of each enzyme (Supplementary Fig. S8). The half-lives of *Th*CE at 8, 25, and 35 °C were 96.3, 3.5, and 0.7 d, respectively. The half-lives of *Cj*CE at 8, 30, and 40 °C were 72.2, 16.0, and 1.0 d, respectively. Compared to the two mesophilic CEs from *R. intestinalis* (with a half-life of 6.3 d at 35 °C) [20] and *T. brennaborense* (with a half-life of 3.8 d at 40 °C) [31], *Th*CE and *Cj*CE exhibit lower thermal stability. However, both *Th*CE and *Cj*CE exhibited good stability at 8 °C, indicating their suitability for low-temperature applications.

**Table 3:** Half-lives of *Th*CE and *Cj*CE (d = day) at different temperatures

Temperature [°C]	*Th***CE**	*Cj***CE**
8	96.3 d	72.2 d
25	3.5 d	-
30	-	16.0 d
35	0.7 d	-
40	-	1.0 d

### Investigation of the epi- and isomerization activity

The epimerization and isomerization activities of the two CEs were measured using 150 mM lactose at the respective maximum temperatures (35 °C for *Th*CE and 40 °C for *Cj*CE) and at 8 °C. As shown in Table 4, it was found that *Th*CE exhibited a specific epimerization activity of 5.95 × 10^2^ ± 4.4 nkat/mg protein at 35 °C, which was approximately 6 times higher than that at 8 °C (1.07 × 10^2^ ± 1.6 nkat/mg protein). Similarly, *Cj*CE showed an epimerization activity of 8.87 × 10^2^ ± 3.9 nkat/mg protein at 40 °C, and a 10 times lower activity at 8 °C (87.8 ± 3.9 nkat/mg protein). Interestingly, *Cj*CE also exhibited isomerization activity at both 40 and 8 °C, with the activity at 40 °C (0.29 ± 1.3 × 10^−3^ nkat/mg) being 170 times higher than that at 8 °C (1.7 × 10^−3^ ± 1.0 × 10^−5^ nkat/mg). By contrast, *Th*CE displayed a low isomerization activity of 0.19 ± 3.7 × 10^−3^ nkat/mg at 35 °C, whereas no activity was detected at 8 °C.

**Table 4:** Specific activities of *Th*CE and *Cj*CE at different conditions

Enzyme	*Th*CE		*Cj*CE	
T [°C]	35	8	40	8
pH [-]	7.5	7.5	9	9
150 mM lactose				
Epi [nkat/mg]^a^	5.95 × 10^2^ ± 4.4	1.07 × 10^2^ ± 1.6	8.87 × 10^2^ ± 3.9	87.8 ± 3.9
Iso [nkat/mg]^b^	0.19 ± 3.7 × 10^−3^	ND^c^	0.29 ± 1.3 × 10^−3^	1.7 × 10^−3^ ± 1.0 × 10^−5^
400 mM lactose				
Epi [nkat/mg]^a^	7.96 × 10^2^ ± 3.0	/^d^	1.37 × 10^3^ ± 5.1	/^d^
Iso [nkat/mg]^b^	41.7 ± 0.7	/^d^	47.5 ± 0.1	/^d^
600 mM lactose				
Epi [nkat/mg]^a^	9.25 × 10^2^ ± 2.4	/^d^	1.69 × 10^3^ ± 2. 7	/^d^
Iso [nkat/mg]^b^	70.8 ± 2.7	/^d^	66.1 ± 0.3	/^d^

^a^ “Epi” means specific epimerization activity

^b^ “Iso” means specific isomerization activity

^c^ “ND” means activity was not detectable (below the calibration range)

^d^ “/” means the activity was not determined

To further characterize the enzymatic properties, the Michaelis constant (*K*_m_) was determined for the epimerization of lactose using nonlinear regression. As shown in Table 2, the *K*_m_ value of *Cj*CE (524.0 mM) was 2.1-fold higher than that of *Th*CE (247.3 mM), indicating that *Th*CE has a better affinity for lactose. Compared to most other reported mesophilic CEs, the results suggest that *Th*CE has a moderate affinity for lactose, whereas *Cj*CE has a relatively low affinity [31]. Notably, it was observed during the determination of the *K*_m_ that the concentration of the isomerized product, lactulose, increased with the increasing substrate concentration of lactose. Based on this observation, the isomerization activity was investigated further at higher substrate concentrations (400 and 600 mM) and at their respective maximum temperatures. When the lactose concentration was increased from 150 to 400 and 600 mM, the isomerization activities of both CEs increased significantly, achieving maximum activities of 70.8 ± 2.7 nkat/mg for *Th*CE and 66.1 ± 0.3 nkat/mg for *Cj*CE with 600 mM lactose (Table 4). In contrast, the epimerization activities of *Th*CE and *Cj*CE increased only to approximately 1.6-fold and 1.9-fold, respectively, when the lactose concentration was increased from 150 mM to 600 mM under their respective temperature maximum (Table 4). Since the reaction rate typically increases rapidly at low substrate concentrations when active sites are unsaturated [32], these results suggest that 400 and 600 mM do not represent saturating concentrations of the substrate lactose. However, concentrations higher than 600 mM could not be tested due to the solubility limit of lactose at room temperature, which has been reported to be approximately 20 g of anhydrous lactose per 100 g of water [33].

### Investigation of the substrate selectivity of *Th*CE and *Cj*CE

To analyze the substrate selectivity of *Th*CE and *Cj*CE, various putative substrates (final concentration of 150 mM) were tested by analyzing the reaction products after incubation for 48 h. Possible substrates were chosen based on the CE catalytic activity, acting on the C2 position of the glucose moiety at the reducing end of *β*-1,4-linked oligosaccharides. As shown in Table 5, neither of the two CEs demonstrates catalytic activity with N-acetylglucosamine as a substrate. This substrate characteristic is consistent with that reported for all CEs [1]. Furthermore, both *Th*CE and *Cj*CE catalyze the conversion of mannose to glucose and glucose to mannose. The concentration of the product glucose after 48 h of reaction was approximately 3.7 times higher than that of the product mannose. However, neither *Th*CE nor *Cj*CE were active for fructose. Regarding disaccharide substrates, both CEs can react on lactose, epilactose, cellobiose, and maltose. Specifically, epimerization and isomerization products were detected when using the substrates lactose and epilactose. The concentration of epimerization products was higher than that of isomerization products. This indicates that the epimerization reaction happened much faster than the isomerization reaction. As a comparison, no products (below the detection limit) were detected after 48 h of incubation of CEs with lactulose, which may be attributed to their low affinity for lactulose. Both *Th*CE and *Cj*CE converted cellobiose into a single product, and it might be the epimer 4-O-*β*-D-glucopyranosyl-D-mannose or the isomer 4-O-*β*-D-glucopyranosyl-D-fructose. Both CEs converted maltose into a single product. It was assumed to be 4-O-*α*-D-glcopyranosyl-D-mannose, since several studies have shown that maltose can be converted into the sole product 4-O-α-D-glucopyranosyl-D-mannose using *Dt*CE and *St*CE [12, 13].

**Table 5:** Substrate selectivity of *Th*CE and *Cj*CE.

Substrate	Product	*Th***CE**	*Cj***CE**
		Product concentration [mM]	
GlcNAc^a^	-^b^	ND^c^	ND^c^
Glc^a^	Man	13.3 ± 0.1	33.5 ± 0.1
Man^a^	Glc	48.6 ± 0.0	122.9 ± 0.7
Fru^a^	-^b^	ND^c^	ND^c^
Lac^a^	Epi	44.6 ± 0.7	45.5 ± 0.0
	Lactu	3.6 ± 0.2	15.6 ± 0.1
Epi^a^	Lac	92.8 ± 0.6	85.7 ± 0.3
	Lactu	3.0 ± 0.3	9.7 ± 0.1
Lactu^a^	-^b^	ND^c^	ND^c^
Cello^a^	4-O-*β*-D-glc-man^d^/ 4-O-*β*-D-glc-fru^d^	+^e^	+^e^
Malto^a^	4-O-*α*-D-glc-man^d^	+^e^	+^e^

^a^ GlcNAc, Glc, Fru, Man, Lac, Epi, Lactu, Cello, and Malto represents N-acetylglucosamine, glucose, fructose, mannose, lactose, epilactose, lactulose, cellobiose, and maltose, respectively

^b^ indicates there is no product shown

^c^ indicates the product concentration is not detectable (lower than the calibration range)

^d^ 4-O-*β*-D-glc-man, 4-O-*β*-D-glc-fru, and 4-O-*α*-D-glc-man means 4-O-*β*-D-glucopyranosyl-D-mannose, 4-O-*β*-D-glucopyranosyl-D-fructose and 4-O-*α*-D-glucopyranosyl-D-mannose, respectively. They are all deduced theoretical products

^e^ “+” represents the formation of the products; the numerical value was not shown due to the absence of the standard substance

By comparison, *Cj*CE demonstrated a stronger catalytic activity toward glucose and mannose than *Th*CE. It exhibited significantly higher catalytic activity than *Th*CE for the isomerization of lactose and epilactose. On the other hand, the catalytic activities of *Th*CE and *Cj*CE were found to be comparable during the epimerization of lactose and epilactose. Overall, *Th*CE exhibited the strongest catalytic capability of converting epilactose (150 mM) to lactose (92.8 ± 0.6 mM), while *Cj*CE exhibited the strongest catalytic capability of converting mannose (150 mM) to glucose (122.9 ± 0.7 mM).

## Conclusion

The genes encoding two putative CEs, one from *T. haidensis* (*Th*CE) and one from *C. japonicus* (*Cj*CE), were cloned and expressed in *E. coli*. The enzymes were then purified and characterized. This study shows that both CEs are mesophilic enzymes that demonstrate considerable epimerization activity at low temperatures (8 °C). Additionally, *Th*CE and *Cj*CE are capable of catalyzing isomerization reactions of lactose at their temperature maximum. Notably, *Cj*CE also exhibited detectable isomerization activity at 8 °C, suggesting its potential for further exploration in low-temperature dairy processing applications. Further studies focusing on the enzymatic conversion of milk lactose at low temperatures could provide valuable insights into its practical utility.

## Electronic supplementary material

Below is the link to the electronic supplementary material.

Supplementary material 1

## Data Availability

The four CE gene sequences analyzed in this study are available in the NCBI nucleotide database under accession numbers [WP_075184903.1] (*Th*CE, from *Teredinibacter haidensis*), [WP_012485926.1] (*Cj*CE, from *Cellvibrio japonicus*), [WP_015048640.1] (*Sa*CE, from *Simiduia agarivorans*), and [WP_007103104.1] (*Pp*CE, from *Paraglaciecola polaris*). No additional raw sequencing data were generated in this study. All other data supporting the findings are available within the article and its supplementary materials. Further detailed information can be made available on request.

## Abbreviation

CE: cellobiose 2-epimerase
*Th*CE: *Teredinibacter haidensis* cellobiose 2-epimerase
*Cj*CE: *Cellvibrio japonicus* cellobiose 2-epimerase
*Pp*CE: *Paraglaciecola polaris* cellobiose 2-epimerase
*Sa*CE: *Simiduia agarivorans* cellobiose 2-epimerase
*Cs*CE: Caldicellulosiruptor saccharolyticus cellobiose 2-epimerase
*Ra*CE: *Ruminococcus albus* cellobiose 2-epimerase
*Dt*CE: Dictyoglomus turgidum cellobiose 2-epimerase
*St*CE: *Spirochaeta thermophila* cellobiose 2-epimerase
*Fj*CE: *Flavobacterium johnsoniae* cellobiose 2-epimerase
*Roin*-CE: *Roseburia intestinalis* cellobiose 2-epimerase
*TrBr*-CE: *Treponema brennaborense* cellobiose 2-epimerase
*Dg*CE: *Dysgonomonas gadei* cellobiose 2-epimerase
ORF: open reading frame
PCR: polymerase chain reaction
*E. coli*: *Escherichia coli*
LB medium: Luria-Bertani medium
2YT: Double yeast extract tryptone
IPTG: Isopropyl-*β*-D-thiogalactopyranosid
OD_600nm_: Optical density at 600nm
IMAC: Immobilized metal ion affinity chromatography
CV: Column volume
SDS-PAGE: Sodium dodecyl sulfate-polyacrylamide gel electrophoresis
SEC: Size exclusion chromatography
HPLC: High-performance liquid chromatography
NCBI: National center for biotechnology information
BLAST: Basic Local Alignment Search Tool
Uniprot: Universal Protein Resource Knowledgebase
