# Supplementary material for: Characterization of novel cellobiose 2-epimerases from Teredinibacter Haidensis and Cellvibrio Japonicus
Source: BMC Biotechnol. 2026 Jun 18;26:79. doi: 10.1186/s12896-026-01184-4 (PMC13281645; doi:10.1186/s12896-026-01184-4)
Supplement: Supplementary file 1 — Supplementary material 1 [file 12896_2026_1184_MOESM1_ESM.docx]

**Supplementary material**

**Characterization of novel cellobiose 2-epimerases from** ***Teredinibacter haidensis* and *Cellvibrio japonicus***

Yaxian Liu^a^, Sabine Lutz-Wahl^a^, Lutz Fischer^a^*

*^a^Institute of Food Science and Biotechnology, Department of Biotechnology and Enzyme Science, University of Hohenheim, Garbenstr. 25, 70599 Stuttgart, Germany*

*Corresponding author: Prof. Lutz Fischer ([lutz.fischer@uni-hohenheim.de](mailto:lutz.fischer@uni-hohenheim.de))

**Codon-optimized nucleotide sequence of** ***Teredinibacter haidensis cellobiose 2-epimerase* (*Th*CE)**

ATGAGCGAACATCTGATTAGCGAAATTCGTGCAGAACTGCATGAAATTGCAGATTGGTGGCTGGTTAATACACCGGATGAAGAAAATGGTGGTTTTATTGGTGAAATCAGCGTGGATAACGTGAAACAGTTTAATGCCGATAAAGGCATTATTCTGAATACCCGCATTCTGTGGTTTTTTAGCGAAGCAGCACTGTTTACCGGCAAAGAAGATTATAAACTGGCAGCAGAACGTGCCTATGCATATCTGACCGAAAAATTCTTTGATAACGAACACGGTGGTGTGTATTGGGCATTAGATGCAACCGGTAAATGTGTGAATGATCGCAAACAAATTTATGCACAGGCCTTTGCAATTTATGGTCTGAGCGCATTCTATAAACTGACCCAGAATGAAGAAGCACTGGAAAAAGCAATGGCGATCTTTGAACTGATTGAAACCCGTGGTCATGATGATGAACGTGGTGGTTATCTGGAAGCATTTGGTCGTGAATGGCAGCCGCTGGAAGATATGCGTCTGAGTGATAAAGATCTGAATAGCCCGAAAAGCATGAATACCCATCTGCATATTCTGGAAGGTTATACCGCACTGTATGTTGCAAATCCGAGTGATGTTGTTGCCAATGCAATGAAACGTTGCCTGGGTTATTTTGATACCCAGATCATCAACAAAGAAAACAACCATCTGCGTATGTTCCAGAGCATGGATTGGAGCGATATGAGCACCAGCATTAGCTATGGCCATGATATTGAAAGCAGCTGGCTGATTTGGGAAGCAGTTGAAGCCCTGGGTGATGAAGCGCTGGAAGCACATTATCGTCCGATTATTCTGGCAATGGCAAAAACCATTCTGGAACAAGGTATTGGTGCAAATGGTGAAGTTCTGGATGCCTATAACTTTGAAACCAGCACACTGCTGGATGAACGCGTTTGGTGGGTTCAAGCAGAAGCAATGGTTGGCTTTCTGAATGCGTATGAACTGACCGATGAAAAAGCATATTATACCGCCTTTGAAAATGCTTGGAGCTTCATCAAACAGTATCAGAAAGATAATGAAAACGGCGAATGGCATTGGCTGAGCACCCTGGATAAACCGCATGTTGGTGATTGTAAAATCGGCTTTTGGAAAGCACCGTATCATAATGGTCGTGCAATGATGGAAGTGTGCAAACTGTTTGTTAAACTGGCCGAAGAATAA

**Amino acid sequence of *Teredinibacter haidensis cellobiose 2-epimerase* (*Th*CE)**

MSEHLISEIRAELHEIADWWLVNTPDEENGGFIGEISVDNVKQFNADKGIILNTRILWFFSEAALFTGKEDYKLAAERAYAYLTEKFFDNEHGGVYWALDATGKCVNDRKQIYAQAFAIYGLSAFYKLTQNEEALEKAMAIFELIETRGHDDERGGYLEAFGREWQPLEDMRLSDKDLNSPKSMNTHLHILEGYTALYVANPSDVVANAMKRCLGYFDTQIINKENNHLRMFQSMDWSDMSTSISYGHDIESSWLIWEAVEALGDEALEAHYRPIILAMAKTILEQGIGANGEVLDAYNFETSTLLDERVWWVQAEAMVGFLNAYELTDEKAYYTAFENAWSFIKQYQKDNENGEWHWLSTLDKPHVGDCKIGFWKAPYHNGRAMMEVCKLFVKLAEE

**Supplementary Fig. S1.** Codon-optimized nucleotide and corresponding amino acid sequences of CE from *Teredinibacter haidensis* (*Th*CE).

**Codon-optimized nucleotide sequence of *Cellvibrio japonicus cellobiose 2-epimerase* (*Cj*CE)**

ATGAGCGCACTGAGCCTGCCGATTCAGAGTCTGACCCTGCAGCAAGAATTTCGTGCAGAACTGATTGCAATTGCCGATTGGTGGGCAACCTATACCATTGATGAAACCCATGGTGGTTTTCATGGTGAAATTACCGCAGATAATCAGCCGGTTGCAAATGCAAGCAAAGGCATTATTCTGAATGCACGCATTCTGTGGTTTTTTAGCGAAGCAGCACAGGTTGTTGATAATCCGCTGTATCGTCGTTGTGCAGAACGTGCATACGATTACCTGCGTAACTATTTTTTCGATCGTGATCATGGTGGTGTGTATTGGGAATTAGATGTGACCGGTAAACCGATCAATACCAAAAAACAGGTTTATGCACAGGCCTTTACCATTTATGCACTGTGTGCATATTTCCAGCTGACCGGTGATGCAGCAGCAGTTGAACAGGCACTGGCATGTTTTAAACTGCTGGAAACACATGCAATTGATCGCGAACATGAAGGTTATCTGGAAGCATTTACCCGTGAATGGGGCACCATTGCAGATGTTCGTCTGAGCGAAAAAGATCTGAATTATCCGAAAAGCCAGAACACCCATCTGCATGTTCTGGAAGCCTATACCACACTGTATCAGGCACATCCGGGTAATGAAGTTCGTGAAGCACTGCGTTATAACATCGAACTGTTTGACAAATACATGATCGATCGCAACACCTATCATCTGCGTATGTTTATGGATCTGGATTGGAAAGATCATTCACCGGGTTTTACCTATGGCCATGATATTGAAGCAAGCTGGCTGATTGCCAAAGCACTGGAAAGCCTGCAGGATGCAGAATATAGCGCACGTCTGACCCCGACACTGATTCGTATTGCAGAAGTTACCGCAGCCGAAGCAATTGGTGAACATGGTCATGTGCTGGATGCGTATGATTTTGCCAGCAAAACCATTAGTCCGGATATTGTTTGGTGGGTTCAAGCAGAAGCAGTTGTTGGTTTTCTGTATGCCTATGCAACCACAGGTGATGAAAAATTCTATCGTGTTGCAGAAACCATCTGGCGTTTTATTCAGCAGTATCAGATTGATCATGAACATGGCGAATGGCTGTGGCTGAGCACCCTGGATGCAGCACGTGCCGAACCGTATTACAAAGTTGGTTTTTGGAAATGCCCGTATCATAATGGTCGTGCAATGATTGAAGCAGTTCGTTACCTGGAAGCCAGCGCAGCCCGTACCGAAAAACATCATAATAATCAGTAA

**Amino acid sequence of *Cellvibrio japonicus cellobiose 2-epimerase* (*Cj*CE)**

MSALSLPIQSLTLQQEFRAELIAIADWWATYTIDETHGGFHGEITADNQPVANASKGIILNARILWFFSEAAQVVDNPLYRRCAERAYDYLRNYFFDRDHGGVYWELDVTGKPINTKKQVYAQAFTIYALCAYFQLTGDAAAVEQALACFKLLETHAIDREHEGYLEAFTREWGTIADVRLSEKDLNYPKSQNTHLHVLEAYTTLYQAHPGNEVREALRYNIELFDKYMIDRNTYHLRMFMDLDWKDHSPGFTYGHDIEASWLIAKALESLQDAEYSARLTPTLIRIAEVTAAEAIGEHGHVLDAYDFASKTISPDIVWWVQAEAVVGFLYAYATTGDEKFYRVAETIWRFIQQYQIDHEHGEWLWLSTLDAARAEPYYKVGFWKCPYHNGRAMIEAVRYLEASAARTEKHHNNQ

**Supplementary Fig. S2. Codon-optimized n**ucleotide and corresponding amino acid sequence of CEs from *Cellvibrio japonicus* (*Cj*CE).

**Codon-optimized nucleotide sequence of *Simiduia agarivorans cellobiose 2-epimerase* (*Sa*CE)**

ATGGCCTGTGAACAAGAACTGGAAAATATTCTGGCATGGTGGCGTACCCATACACTGGATAGCGAAGGTTTTGTTGCAGAACTGGATAACAATGGTCAGCGTAATGTTCATGCCGAAAAAGGCATTATTCTGAATACCCGTATCCTGTGGTTTTTTAGCGAACTGGCAATTCAGCGTCCGGCACTGGAATGTGGTGATCTGGCAGATCGTGCATTTGATTATCTGACCGATCACTTTTTCGATGAAGAATATGGTGGTCTGTTTTGGAGCCTGGATGCAGCAGGTCAGATGTGTGGTGATAAAAAACAGACGTATGCACAGGCCTTTGGTATTTATGCACTGAGCGCATATTATCGCCTGACCAAAAAACCGGAAGCACTGGCATTTGCAATGGACCTGTTTCGTCTGATTGAACAGCATTGTCTGGATCGTGCAAGCGGTGGTTATGTTGAAGCAAAAAGCCGTCAGTGGGAACCGCTGGTTGATGTTCGTCTGAGCGCAAAAGATGATAATGCACCGAAAACCATGAACAACCATCTGCATGTTCTGGAAGCATATACCGGTCTGTATCTGGCAAATCCGACACGTGAAACCGAACAGGCACTGCGTAATAACATTCTGTGGATGTGTGAACGTATTGCAGATGCAGATACAGGTCATCTGAAACTGTTTCTGGATATGCAGTGGAATGATCACAGCAGCTGTTATAGCTATGGCCATGATATTGAAGCAAGCTGGCTGATTTGTGAAGCCCTGGAAGTTCTGGGTGATGAAGTTCTGCTGAATCGTTTTAGCCCTCTGGTTGTTAATCTGGCCAAAACCTGTCTGGCCGAAGGTATTGGTGAACATGGTCAGGTTCTGGATAAATTCGATAAAACCAGTGGTGAACGTCATCCGGAAAGCGAATGGTGGGTTCAAGCAGAAGCAATGGTTGGTTTTGTTAATGCATGGCAGCTGACCGGTGATGAAAAATTTCTGCGTGCAACCGAAGCAGTTTGGGTTTATATTCAGCAGTATCAGCTGGATAAAAACCTTGGTGAATGGTTTTGGTATAGCACCCTGGATCAGGCACGTGGTCATCAGCACTATAAAATGGGTTTTTGGAAAGCACCGTATCATAATGGTCGTGCAATGCTGGAAGTTGCAAATCGTCTGAGTGCAGCCTGTCAGAAAAGCGAATAA

**Amino acid sequence of *Simiduia agarivorans cellobiose 2-epimerase (SaCE)***

MACEQELENILAWWRTHTLDSEGFVAELDNNGQRNVHAEKGIILNTRILWFFSELAIQRPALECGDLADRAFDYLTDHFFDEEYGGLFWSLDAAGQMCGDKKQTYAQAFGIYALSAYYRLTKKPEALAFAMDLFRLIEQHCLDRASGGYVEAKSRQWEPLVDVRLSAKDDNAPKTMNNHLHVLEAYTGLYLANPTRETEQALRNNILWMCERIADADTGHLKLFLDMQWNDHSSCYSYGHDIEASWLICEALEVLGDEVLLNRFSPLVVNLAKTCLAEGIGEHGQVLDKFDKTSGERHPESEWWVQAEAMVGFVNAWQLTGDEKFLRATEAVWVYIQQYQLDKNLGEWFWYSTLDQARGHQHYKMGFWKAPYHNGRAMLEVANRLSAACQKSE

**Supplementary Fig. S3.** Codon-optimized nucleotide and corresponding amino acid sequence of CE from *Simiduia agarivorans* (*Sa*CE).

**Codon-optimized nucleotide sequence of *Paraglaciecola polaris (PpCE)***

ATGAGCCTGGCAGCAACCCATGCAATTCTGACCACACCGCTGAATCGTCAGAGCGTTGAAGGTGAACTGACCCATATTGCAGATTGGTGGGTTAAACATAGCGTGGATCATGTTAATGGTGGCTTTTATGGCGAAATTGATTTTGTTGCACAGCCGCTGAGTGATGCAAATAAAGGTGTTATTCTGAATAGCCGCATCCTGTGGTTTTTTAGCGAAATGGCACTGAAAGATGATAGCGCACAGTATAAAACCCTGGCAATTCGTGCATTTGAATATCTGCTGGCACACTTCGATGATAAAGAACATGGTGGTGCATATTGGGAAGTTGCATTTGATGGTCGTCTGCTGCAGGGTAAAAAACAGACCTATGCACAGTGCTTTTGTATTTATGCACTGTGTAGCTATTTTCGCCTGACCGCAGATCCGCTGGCACTGGAAAAAGCAGTGAGCTATTTTAACCTGGTTGAACAGAATGCCCGTGATGTTAAATGTGGTGGTTATATTGAAGCCTGTAGCGAAGATTGGAGCAGCATTAGCGATTATCGTCTGAGCGATAAAGATCTGAATTTCCCGAAAAGCATGAACACCCATCTGCATGTTCTGGAAGCATATAGTGCACTGTATCAGGTTCATAAAACCGCACAGACCGAAGAAGCACTGCGTCATATTATTGATGTGTTTCAGTTTCACATCATCTGCCACAAAAGCGCACATCTGAAACTGTTTTTTGATATGCAGTGGCGTGATCAGAGCCAGACCTATAGCTTTGGTCATGATATCGAAGCAAGCTGGCTGCTGTGGGAAAGCGCATGTGTTCTGGGTGATAAACGTGTTATGGGTAAACTGAAGCCGATCATTATTGATCTGGCACGTGCATGTCTGGATGAAGCAATTGGTGATGCAGGTCAGGTTTGTGATGAATTTATCTTTAGCAGCCGTAAACGTGTGTCAACCAGCTACTGGTGGGTTCAAGCAGAAGCCCTGGTTGGCTTTATGAATGCATTTGCACTGACCAAAGACGAGAAATACAAAGCAGCATGTCAGCCGATTTGGGCATTTATTCAGCAGTATCACATTGATAGCGTTCATGGTGAATGGCATTGGCTGGCAAGCCAGGATCAGGATACCGATAGCCGTATCTATAAAGCAGGTTTTTGGAAAGCACCGTATCATAATGGTCGTGCAATGATGGAACTGCAGCGTCTGCTGGGTCAGAGCTAA

**Amino acid sequence of *Paraglaciecola polaris (PpCE)***

MSLAATHAILTTPLNRQSVEGELTHIADWWVKHSVDHVNGGFYGEIDFVAQPLSDANKGVILNSRILWFFSEMALKDDSAQYKTLAIRAFEYLLAHFDDKEHGGAYWEVAFDGRLLQGKKQTYAQCFCIYALCSYFRLTADPLALEKAVSYFNLVEQNARDVKCGGYIEACSEDWSSISDYRLSDKDLNFPKSMNTHLHVLEAYSALYQVHKTAQTEEALRHIIDVFQFHIICHKSAHLKLFFDMQWRDQSQTYSFGHDIEASWLLWESACVLGDKRVMGKLKPIIIDLARACLDEAIGDAGQVCDEFIFSSRKRVSTSYWWVQAEALVGFMNAFALTKDEKYKAACQPIWAFIQQYHIDSVHGEWHWLASQDQDTDSRIYKAGFWKAPYHNGRAMMELQRLLGQS

**Supplementary Fig. S4.** Codon-optimized nucleotide and corresponding amino acid sequence of CE from *Paraglaciecola polaris* (*Pp*CE).


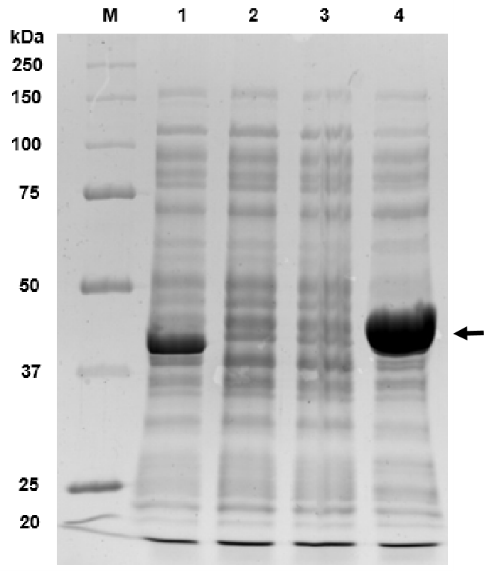


**Supplementary Fig. S5.** SDS-PAGE analysis of recombinant CEs after induction for 5 h in *E. coli* BL21(DE3). M: Precision Plus Protein™ unstained protein standard (10 ~ 250 kDa, Bio-Rad laboratories GmbH); 1: *Th*CE; 2: *Simiduia agarivorans* CE; 3: *Paraglaciecola Polaris* CE; 4: *Cj*CE (the black arrow indicates the position of the CE band).


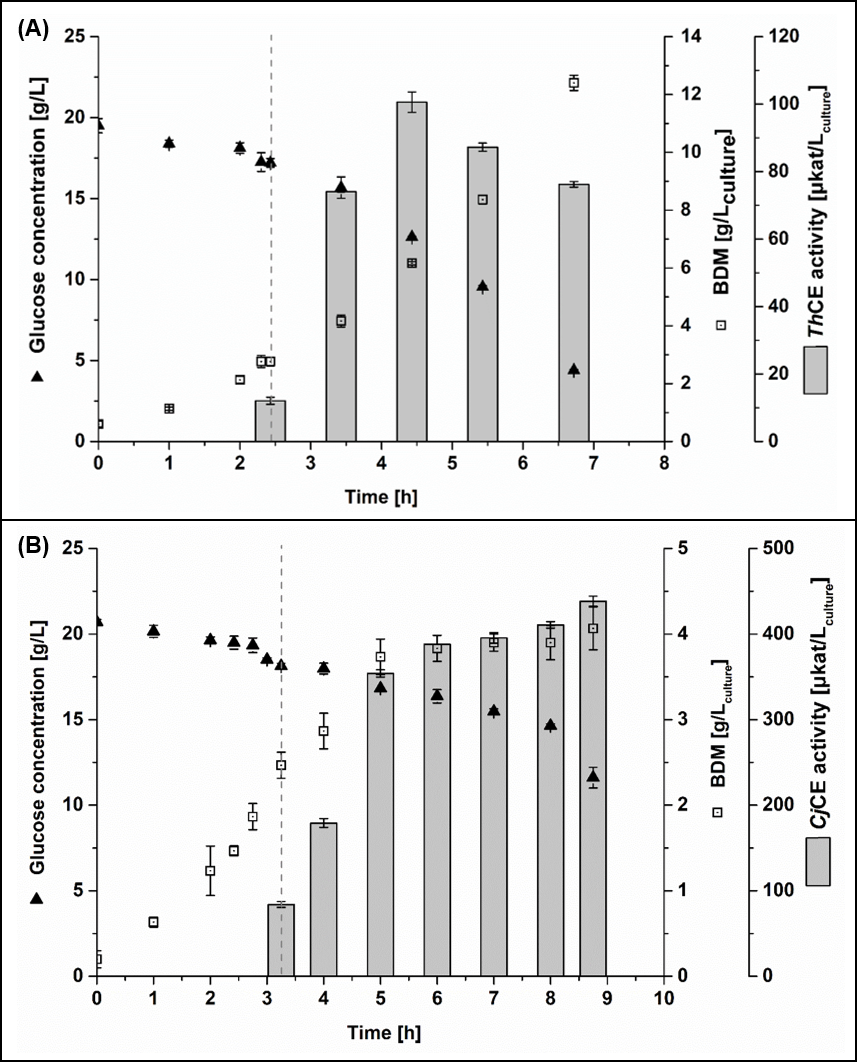


**Supplementary Fig. S6.** Bioreactor cultivation of the recombinant *E. coli* BL21 (DE3) for the production of the CEs: (A) *Th*CE and (B) *Cj*CE. Working volume 5 L. Dash line indicates the induction with IPTG and temperature shift from 37 to 30 °C.


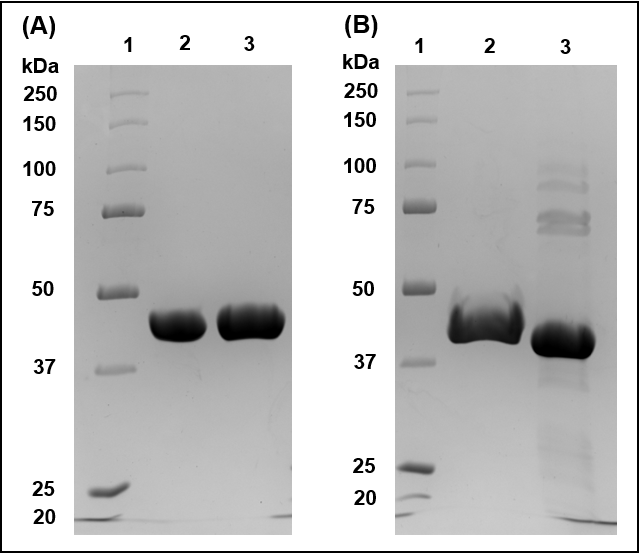


**Supplementary Fig. S7.** The SDS-PAGE analysis of samples taken after SEC of the CEs: (A) *Th*CE, (B) *Cj*CE. Lane 1: Precision Plus Protein™ unstained protein standard (10 – 250 kDa, Bio-Rad laboratories GmbH), lane 2: CE after SEC, lane 3: CE before SEC (IMAC purified CE).

**
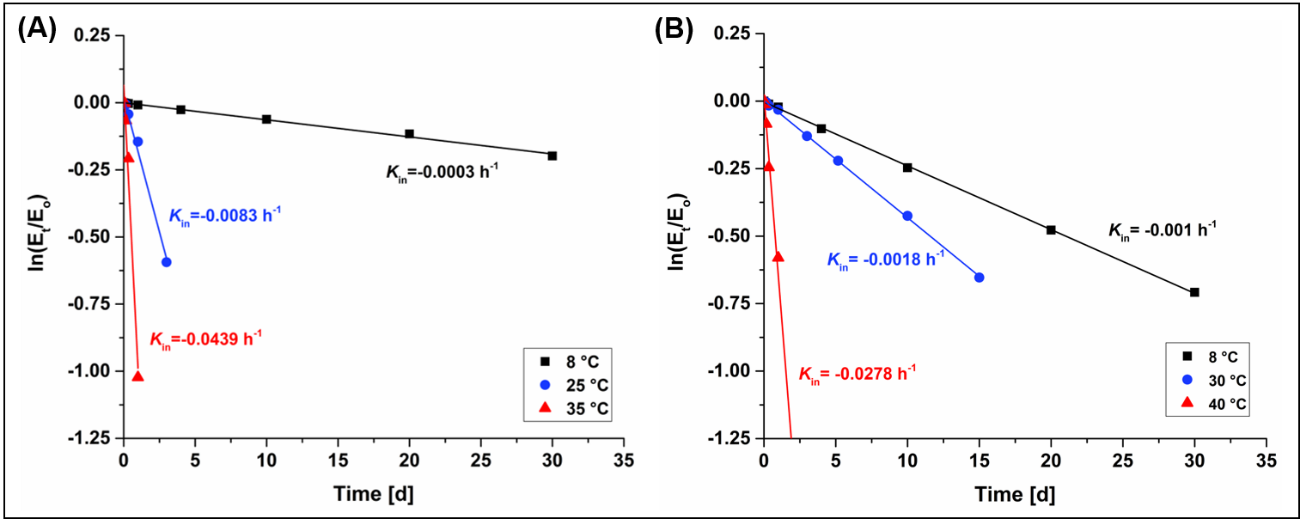
**

**Supplementary Fig. S8.** Inactivation rate constant (*K*_in_) of the CEs at different temperatures. (A) *Th*CE (B) *Cj*CE. The y-axis represents the natural log value of the residual CE epimerization activity divided by the initial CE epimerization activity.

**Supplementary Table 1.** Open reading frame (ORF) templates used for *in silico* screening of novel CEs.

|  | Amino acid sequence |
| --- | --- |
| ORF1^*^  (338 aa) | >ORF1 vineyard soil  MARVFQQDNARILWFFSEVAQEVDNPVYRSCATRAYDYVMTHFFDNEQGGVYWELDAAAVPINTKKQVYAQAFTIYALCTYYQLTRDEQALVRALECFKLVEQYAIDHGREGYLEAFTRDWGVIEDLRLSEKDLNYPKSQNTHLHILEAYTALYQAHPAPEVKAALKYNIQMFDKYMIDKNTHHLRMFMDLEWKDFSPGYTYGHDIEASWLIAKALESLGDSAYNAELTPTLISIAQVTLNEAIGEQGQVIDSFDFSTRRTNVDTVWWVQAEALVGFLYAYATTGEEAFYRAAENCWXFIKRYQIDHXRGXXFWLSSXXKLNADXYXKVGFXKCPYXNG |
| ORF2^**^  (308 aa) | **>** ORF2 cave soil  MIGAFQYVIEYFDDEDNGGVFWELAWDGSLVNSKKQTYAMCFCIYAFVTYYRLTSNNVALAKSLQYFDLIEAHARDREHGGYAEAFARDWRPLDDVRLSAEDMNAPKTMSTHLHLLEAYTALYIGTASDRTHAALRHAIDIFFENIVNHDNGHLNLFFDREWNVLSSTISFGHDIEASWLLWKAGEKLGDTKLLTKLKPIVERLADACHAEGIGDINQVCTKYDPENGRRCEKGVWWVQAEALVGFLNAYQLTGXQRYRTACDEVWRFICKHHIDPVGXEWHWLSTAHXNGXNRNYXTGFWXCPYXNGS |

^*^Putative CE ORF1 was identified from vineyard soil (Esslingen, Germany) metagenome.

^**^Putative CE ORF2 was identified from cave soil (Sonnenbühl, Germany) metagenome.

**Supplementary Table 2.** Primer sequences for amplification of the gene of *Th*CE and *Cj*CE (bases highlighted in red and green indicate the restriction sites of *Nde*I and *Xho*I, respectively).

| Primer | Sequence 5’-3’ |
| --- | --- |
| *Th*CE_for_primer | CTCATATGAGCGAACATCTGATTAGC |
| *Th*CE_rev_primer | GGCTCGAGTTCTTCGGCCAGTTTAACAAAC |
| *Cj*CE_ for_primer | CTCATATGAGCGCACTGAGC |
| *Cj*CE _rev_primer | GTCTCGAGCTGATTATTATGATGTTTTTCGGTACGGGC |
| *Sa*CE_for_primer | GGCATATGGCCTGTGAACAAGAAC |
| *Sa*CE_rev_primer | AACTCGAG TTCGCTTTTCTGACAGGCTG |
| *Pp*CE_for_primer | TTCATATGAGCCTGGCAGCAAC |
| *Pp*CE_rev_primer | GGCTCGAGGCTCTGACCCAGCAGACG |
